# Mechanical loading of isolated cardiac muscle with a real‐time computed Windkessel model of the vasculature impedance

**DOI:** 10.14814/phy2.14184

**Published:** 2019-09-11

**Authors:** Amy S. Garrett, Toan Pham, Denis Loiselle, June‐Chiew Han, Andrew Taberner

**Affiliations:** ^1^ Auckland Bioengineering Institute, The University of Auckland Auckland New Zealand; ^2^ Department of Physiology The University of Auckland Auckland New Zealand; ^3^ Department of Engineering Science The University of Auckland Auckland New Zealand

**Keywords:** Arterial Impedance, force‐length loop, heart muscle, mechanical work, Windkessel model

## Abstract

To date, the mechanical loads imposed on isolated cardiac muscle tissue in vitro have been oversimplified. Researchers typically applied loads that are time‐invariant, resulting in either isometric and auxotonic contractions, or flat‐topped (isotonic shortening) work‐loops. These contraction types do not fully capture the dynamic response of contracting tissues adapting to a variable load, such as is experienced by ventricular tissue in vivo. In this study, we have successfully developed a loading system that presents a model‐based, time‐varying, continuously updated, load to cardiac tissue preparations. We combined a Windkessel model of vascular fluid impedance together with Laplace's Law and encoded it in a real‐time hardware‐based force‐length control system. Experiments were carried out on isolated rat left ventricular trabeculae; we directly compare the work‐loops arising from this protocol with those of a typical simplified isotonic shortening work‐loop system. We found that, under body conditions, cardiac trabeculae achieved greater mechanical work output against our new loading system, than with the simplified isotonic work‐loop protocol. We further tested whether loading the tissue with a mechanical impedance defined by “diseased” Windkessel model parameters had an effect on the performance of healthy trabeculae. We found that trabecula shortening decreased when applying the set of Windkessel parameters describing the hypertensive condition, and increased in the hypotensive state. Our implementation of a real‐time model of arterial characteristics provides an improved, physiologically derived, instantly calculated load for use in studying isolated cardiac muscle, and is readily applicable to study various disease conditions.

## Introduction

Cardiac output is determined by the mechanics of the heart, and of the circulation into which it empties. During each heartbeat, the pressure in the ventricles increases until it exceeds that of the arterial vasculature, thus opening the aortic and pulmonary valves, and ejecting blood into their respective circulations. During the ejection period, the pressure in the left ventricle drives against the impedance of the systemic circulation, which is contributed by the compliance of the large arteries proximal to the systemic circulation, and the resistance of smaller peripheral arteries. These physical characteristics in turn determine the time‐course of pressure decay in the aorta during diastole, and thus the pressure required to be developed in the left ventricle for ejection to occur during the following beat (Sonnenblick and Downing, 1963; Milnor, 1975).

Arterial pressure and flow thus vary at rates strongly dependent on the elastic properties of the arterial walls, and the resistance to the flow of blood through the arterial system. During systole, when blood is ejected from the left ventricle, the arterial walls distend. This results in the storage of elastic energy, especially in the large, proximal, systemic arteries. During diastole, this pressure discharges, and flow is sustained through the peripheral arteries and arterioles. This process reduces large fluctuations in pressure throughout each cardiac cycle, thereby supplying a consistent flow of blood to the distal systemic vessels and the tissues that they supply (Frank, 1899; Westerhof et al., 2009).

For well over a century (Coats, 1869; Patterson et al., 1914; Frank, 1990; Zimmer, 2002), clinician‐scientists have interrogated the mechanical function of the ex vivo heart by recording its pressure‐volume‐time behavior in response to physiological and pharmacological perturbations. Since such early studies, experimental physiologists have commonly adopted simpler preparations, namely: isolated tissues (Elzinga and Westerhof, 1981; Mellors et al., 2001; Han et al., 2019) or cells (Iribe et al., 2007; Helmes et al., 2016), where force and length become proxies for pressure and volume. In such cases, researchers endeavor to approximate physiological conditions as closely as possible by adopting contraction patterns that mimic those of the heart in vivo. The extremes of such patterns are represented by isometric contractions and unloaded isotonic contractions (either of which can be performed at various muscle lengths).

Situated between these extremes are “work‐loops.” The appeal of such loops is that they commence with a period of isometric force development (mimicking the isovolumic pressure‐development phase of the ventricle in vivo), and are followed by a phase of muscle shortening (mimicking the ejection phase of systole), before the muscle relaxes at shortened muscle length (mimicking the phase of isovolumic relaxation). Under this scenario, experimental approximation of both isovolumic phases has generally been acceptable; that of the shortening phase has not. In contrast to the auxotonic nature of ejection in vivo, in vitro simulations have usually been performed isotonically, thereby producing “flat‐topped” work‐loops (Gibbs et al., 1967; Elzinga and Westerhof, 1981; Helmes et al., 2016; Han et al., 2019). These oversimplified work‐loop protocols only approximately replicate the ejection mechanics of the ventricle.

To overcome this shortcoming, other experimentation on ventricular tissues has been carried out using a Windkessel‐type loading system (Elzinga and Westerhof, 1981; Elzinga and Westerhof, 1982; Tombe and Little, 1994). While this loading technique provides a more realistic time‐varying load than the “isotonic shortening” protocol, it requires the shortening trajectory to be precalculated, and is applied only to a single work‐loop twitch, preceded and followed by isometric twitches. Not only does this method require a priori estimation of the magnitude of the applied load, but it also does not allow the muscle to adapt in real‐time to changes in the mechanical impedance against which it is contracting. The resulting response of the muscle thus does not reflect its steady‐state behavior following the change of the load.

In this investigation, we have implemented an experimental technique that overcomes these limitations. Our method uses a high‐speed, hardware‐based, computation of a parameterized Windkessel‐style model of vascular impedance. Every 50 *µ*sec, the force developed by an isolated muscle is presented to the model, resulting in new predictions of arterial pressure and flow rate, and their one‐dimensional equivalents (muscle force and velocity of shortening). The model instantly reacts to changes in force development or model parameters, and immediately determines the mechanical impedance that is then presented to the muscle. Our mechanical loading technique thus allows isolated muscle to experience a parameterized time‐varying load that more closely approximates the load experienced by the tissues within the left ventricle. We imposed this impedance‐based load on isolated ventricular trabeculae and investigated the effects of varying model parameters on the performance of the muscle. For the first time, we were able to track the adaptive response of the muscle upon a change in model parameters, unlike previous studies using fixed‐afterload isotonic protocols (Han et al., 2009; Taberner et al., 2011a,b; Helmes et al., 2016; Han et al., 2017) and the precalculated afterload protocol (Elzinga and Westerhof, 1981; Tombe and Little, 1994; Iribe et al., 2007) as described in the preceding paragraph. With this capability, we then demonstrated the effect of applying “diseased” arterial system load parameters to a healthy muscle sample.

## Methods

### Model description

**Figure 1:**
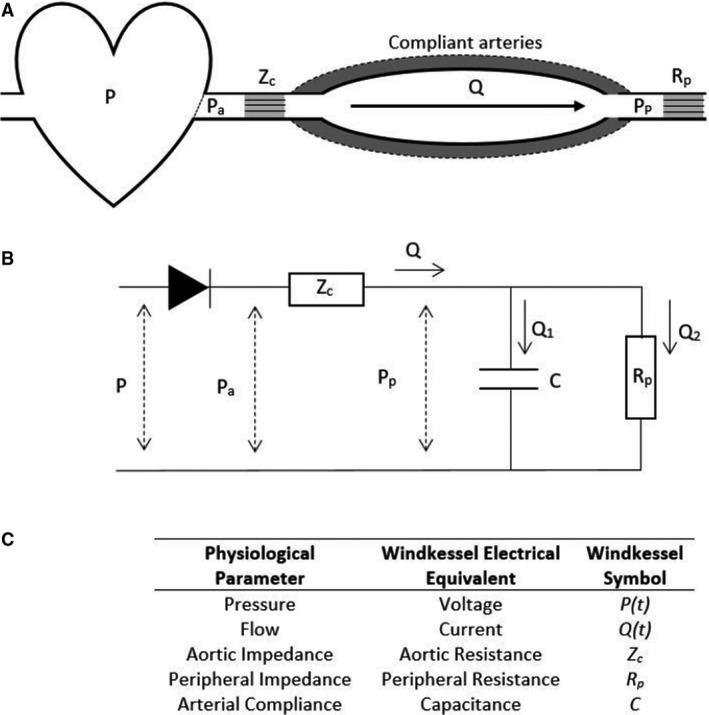
Mechanical and electrical representation of a 3‐element Windkessel system. (A) A diagram of the Windkessel arterial system. (B) A diagram of the electrical circuit representing a 3‐element Windkessel model. (C) Relation of Windkessel parameters to their physiological equivalents.

In this study, we describe the mechanical impedance of the arterial system (summarized by the diagram in Fig. 1A) using a lumped‐parameter Windkessel model, as first proposed by Frank (1899) (English translation by Sagawa et al. (1990). In this formulation, ventricular pressure, aortic flow, and arterial impedance are represented by their electrical circuit equivalents (Westerhof et al., 2009): blood flow, pressure, and hydraulic impedance are analogous to current, voltage, and electrical impedance, respectively (Fig. 1C). Both pressure and flow rate vary with time, and depend upon the input “pulse” delivered to the system. The peripheral resistance of the vasculature and compliance of the large arteries are represented by a resistor (*R_p_*) and capacitor (*C*) in parallel, and the characteristic aortic impedance is represented as a resistor (*Z_C_*) through which flow (*Q*) is developed. The aortic valve is represented as a diode of zero forward voltage (Fig. 1B).

The pressure at various points in the arterial system is denoted as *P* with various subscripts: in the absence of a subscript, *P* denotes the input pressure pulse, *P_a_* denotes the pressure in the aorta, and *P_p_* denotes the peripheral pressure in the systemic circulation. In the analogous electrical circuit, when the input pressure signal, *P*, is large enough to allow current to flow through the diode, charge is stored in the capacitor, mimicking the distension of the large arteries and net increase of blood volume in the proximal arterial system. During diastole, no flow passes through the characteristic impedance (*Z_C_*) and the aortic pressure and peripheral pressure are equal (*P_a_* = *P_p_*). The stored charge in the capacitor discharges through the peripheral resistor, which results in an exponential decay of aortic pressure. This pressure decay occurs at a rate dependent solely on *R_p_* and *C*, referred to as the *RC* time constant (Westerhof et al., 2009).

### Model formulation

**Figure 2:**
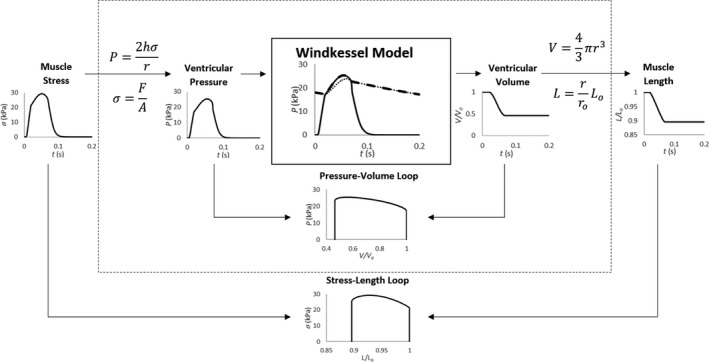
Illustration of the combined Windkessel/Laplace model using simulated data. Muscle stress was converted in real‐time to pressure within the ventricle using Laplace's Law. Using this pressure, the Windkessel model provides an estimate of blood flow rate, and ventricular volume, which is then scaled back to muscle length. In the Windkessel model plot, the dotted line is peripheral pressure (*P_p_*), whereas the broken line is arterial pressure (*P_a_*). The resulting stress‐length loop reflects the equivalent pressure‐volume loop.

In order to apply a Windkessel‐modeled mechanical impedance to a contracting muscle, the equivalent aortic and peripheral pressures and the blood flow leaving the ventricle were computed in real‐time. First, the force produced by a contracting cardiac trabecula was converted into the corresponding blood pressure that would arise within the ventricle. This conversion was achieved using Laplace's Law (Mirsky, 1969; Seymour and Blaylock, 2000) with its assumption that the geometry of the ventricle can be approximated as an elastic spherical chamber. Scaling the force developed by the muscle to an appropriate ventricular pressure allowed the Windkessel model to be expressed and solved in its original form. A diagram of the model structure and computation process is shown in Figure 2, with full details available in Appendix A.

### Software and hardware model implementation

The 3‐element Windkessel/Laplace model was encoded in a LabVIEW (2017) Real‐Time Desktop data acquisition and control system, as described previously (Garrett et al., 2017). Model parameters were provided by the experimenter, together with experimentally determined muscle dimensions (diameter and the muscle length that results in maximum active force, referred to as optimal length or *L_o_*). Estimates of muscle force and muscle length were acquired from a laser interferometer, via a field programmable gate array (FPGA) control system, as described previously (Taberner et al., 2011a; 2015; 2018). Muscle force was converted to wall stress (*σ*) and ventricular pressure (*P*) using trabecula dimensions, and nominal resting dimensions for the rat left ventricle.

The pressure presented to the model depends on the state of the aortic valve, and requires the comparison of its values either side of the valve (*P* and *P_a_*). When the pressure in the ventricle (*P*) is lower than the pressure in the aorta (*P_a_*), the aortic valve remains closed, preventing blood flow (*Q = 0*). Conversely, when the pressure in the ventricle exceeds the pressure in the aorta, the aortic valve opens, thereby allowing the flow of blood into the vasculature. This conditional step determines the pressure input to the Windkessel transfer function, *Z_Wk_* (Equation 5, Appendix A).

Equation 5 was transformed into its discrete time equivalent, and encoded in a 20 kHz deterministic loop using the *Discrete Transfer Function* LabVIEW algorithm. The output of this function provides an instantaneous estimate of the rate of blood flow (*Q*) and hence the change in volume of the ventricle. The change in volume was then converted to muscle length, providing a position set‐point that was immediately presented to the motor control hardware. The pressures arising from the Windkessel model (*P_a_* and *P_p_*) and corresponding dimensions (*V*, *r* and *L*) were fed back as initial conditions for the next loop cycle. The refilling phase of the cardiac cycle was achieved by imposing a user‐selected rate of volume in‐flow initiated during diastole. In this way, the equations relating instantaneous muscle force to muscle length were solved to provide an updated motor position set‐point every 50 *µ*sec.

### Software validation

**Figure 3:**
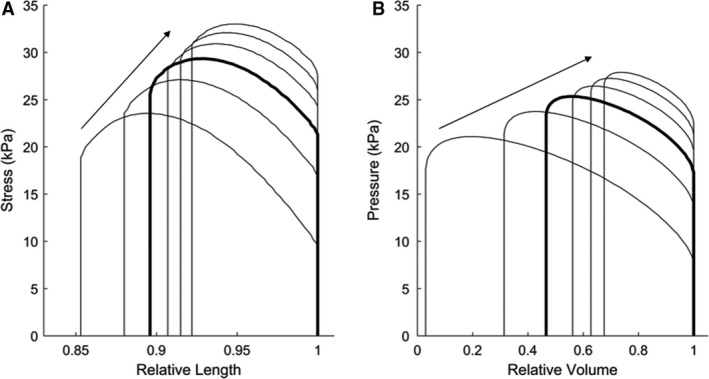
Simulated work‐loops to illustrate the effect of changing peripheral resistance. A series of stress‐length work‐loops (A) and pressure‐volume loops (B) were simulated by increasing the Windkessel parameter for peripheral resistance in the order indicated by the arrows from 5 GPa⋅s⋅m^−3^ to 50 GPa⋅s⋅m^−3^. The control “normotensive” Windkessel parameters result in the bold work‐loops (also shown in Fig. 2).

Prior to conducting experiments on a contracting cardiac trabecula, simulations were performed to validate the computation of model solutions, and demonstrate the robustness of the model output as Windkessel parameter values were varied. For these “dry run” simulations, a previously recorded twitch force developed by a trabecula was applied as input to the model. The magnitude of the force twitch was 3.5 mN, corresponding to the isometric twitch magnitude of a trabecula of typical dimensions (*L_o_* = 1.8 mm, diameter = 0.26 mm). The model returned estimates of trabecula shortening in order to simulate work‐loops (Fig. 3A), together with ventricular, arterial, and peripheral pressures.

Figure 3 shows pressure‐volume (PV) loops and work‐loops for normal rat Windkessel parameters (*R_p_* = 14.5 GPa⋅s⋅m^−3^, *C* = 32 pm^3^⋅Pa^−1^⋅sec^−1^, *Z_C_* = 0.5 GPa⋅s⋅m^−3^), indicated by the bold line (Kind et al., 2010). The internal model states of ventricular, arterial, and peripheral pressure for normal Windkessel parameters are displayed in Figure 2. The Windkessel impedance load was modulated by increasing the model value for peripheral resistance (*R_p_*) in the order indicated by the arrows from 5 GPa⋅s⋅m^−3^ to 50 GPa⋅s⋅m^−3^.

These simulations demonstrate the range and shape of work‐loops that arose when model parameters were varied from typical “normal” values. The stress‐length and pressure‐volume loops now exhibited a “realistic” shape that is more reminiscent of pressure‐volume loops recorded in the intact heart (Pacher et al. 2008; Goo et al. 2013). The shapes of the stress‐length and pressure‐volume curves differ from each other because of the cubic relationship between muscle length and chamber volume. Although the input twitch force was prescribed and thus invariant with shortening, these simulations provided validation that the model system functioned as required, and allowed us to investigate the effects of changing model parameters from their normal values. Furthermore, the model output was robustly computed in a timely manner across all values of the Windkessel parameters explored, with no evidence of any numerical instability in model solutions or in internal parameters.

### Experimental procedure

The hearts of male Wistar rats were removed immediately subsequent to the application of isoflurane anesthesia (5% in oxygen) and heparin (1000 IU kg^−1^), and cervical dislocation, in accordance with protocols approved by the University of Auckland Animal Ethics Committee: R1341 (under the aegis of the New Zealand Ministry of Primary Industries). Subsequent procedures conformed to Directive 2010/63/EU of the European Parliament on the protection of animals used for scientific purposes – in particular, Article 5(a): “Procedures carried out for the purpose of basic research”. Each excised heart was promptly Langendorff‐perfused with 100% oxygenated Tyrode solution comprising 130‐mmol/L NaCl, 6‐mmol/L KCl, 1.5‐mmol/L MgCl_2_, 0.5‐mmol/L NaH_2_PO_4_, 0.3‐mmol/L CaCl_2_, 10‐mmol/L glucose, and 20‐mmol/L 2,3‐butanedione monoxime (BDM) and the pH adjusted to 7.4 with Tris. Trabeculae were dissected from the left ventricle. These small muscles are typically 1.5–3 mm long and less than 0.4 mm in diameter. Their small diameter allows for adequate oxygenation during in vitro experimentation, which avoids tissue hypoxia (Han et al., 2011). Dissected trabeculae were transferred to a work‐loop device, and superfused with the same oxygenated Tyrode's solution but in the absence of BDM and at a CaCl_2_ concentration of 1.5 mmol/L. The flow of superfusate was electronically (Taberner et al., 2018) maintained at 0.54 *μ*L sec^−1^.

Force‐length perturbations were performed in the work‐loop device, developed at the University of Auckland and described previously (Taberner et al., 2011a; 2015; 2018). Briefly, a ventricular trabecula is held between two platinum hooks in the center of a bath of 1‐mm square cross section. The right hook attaches to a custom‐built force transducer for measuring the time‐varying force developed by the trabecula during contraction. The left hook attaches to a linear length motor, which can be controlled to allow the trabecula to shorten in order to perform a work‐loop. The position of the motor and the displacement of the force transducer arm are determined by an interferometer‐based measurement system. Analysis of force signals and control of muscle length are computed by a combination of hardware‐ and software‐based control systems in a LabVIEW 2017 FPGA and Real‐Time architecture. Trabecula contraction was initiated by electrical stimulation (typically pulses of 4 V magnitude and 4 msec duration) through a pair of platinum hooks placed upstream and downstream of the muscle.

Optimal length of the trabecula (*L_o_*) was determined by increasing muscle length incrementally until the developed (active) force was maximal. The dimensions of the trabecula (optimal length and orthogonal diameters) were determined using a microscope graticule.

A single trabecula was dissected from three animals for experiments. Each was subjected to different experimental conditions: room temperature (24°C) contractions at 1 Hz, 32°C contractions at 1 Hz, and body temperature (37°C) contractions at 5 Hz. Each combination of stimulus frequency and temperature ensured completion of diastole prior to the next twitch. We chose to perform experiments under these three distinct experimental conditions in order to demonstrate the range of capability of our loading technique.

The experimental protocol applied at 24°C first involved allowing the trabecula to develop isometric twitches. Second, “flat‐topped” work‐loops were performed over the developed force range at six different time‐invariant values of afterload. Finally, Windkessel‐modeled time‐varying loads were applied by changing each of the three Windkessel parameters in turn. Initial values for the three Windkessel parameters were determined from the literature (Molino et al., 1998). The experimental protocol applied at 32°C involved stepping only the Windkessel peripheral resistance, *R_p_*, from an initial low value (50 GPa⋅s⋅m^−3^) to a high value (500 GPa⋅s⋅m^−3^) while the muscle was performing a Windkessel‐loaded contraction at 1 Hz. The experimental protocol applied at 37°C first involved allowing the trabecula to develop isometric twitches. Second, “flat‐topped” work‐loops were performed over the developed force range at six different time‐invariant values of afterload. Windkessel‐modeled time‐varying loads were applied by changing only the peripheral resistance parameter. Finally, all three Windkessel parameters were varied together in order to simulate hypertension and hypotension.

## Results

**Figure 4:**
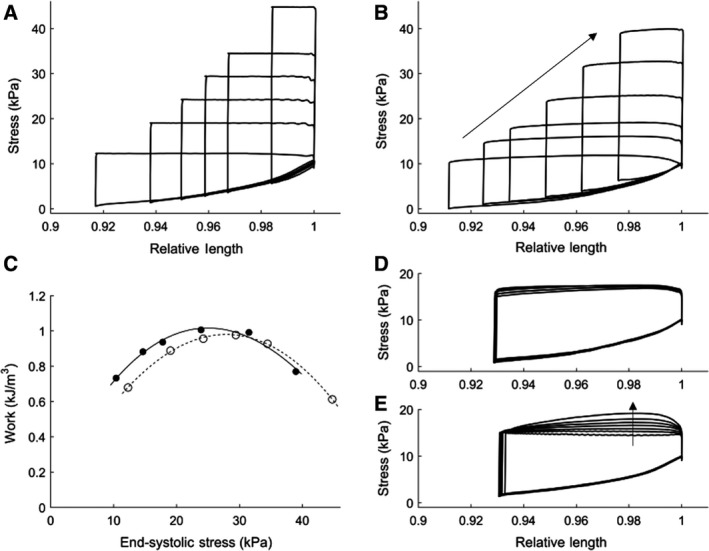
Work‐loop experiments performed at room temperature (24°C) and 1 Hz stimulation. Trabecula dimensions: *L_o_* = 2.77 mm and diameter = 0.364 mm. (A) Flat‐topped work‐loops at a range of user defined constant afterloads. (B) Windkessel‐loaded work‐loops obtained by increasing peripheral resistance from 50 GPa⋅s⋅m^−3^ to 2 TPa⋅s⋅m^−3^ in the direction indicated by the arrow. The base Windkessel parameter values were: *R_p_* = 150 GPa⋅s⋅m^−3^, *C* = 32 pm^3^⋅Pa^−1^⋅sec^−1^, *Z_c_* = 5 GPa⋅s⋅m^−3^. (C) Work performed by the trabecula during flat‐topped (unfilled) and Windkessel‐loaded (filled) work‐loops. Polynomial lines of best fit through each dataset are constrained at the origin. (D) Windkessel‐loaded work‐loops when only *C* was changed from 15 pm^3^⋅Pa^−1^⋅sec^−1^ to 80 pm^3^⋅Pa^−1^⋅sec^−1^. (E) Windkessel‐loaded work‐loops when only *Z_C_* was increased from 0.5 to 15 GPa⋅s⋅m^−3^, as indicated by the arrow. One muscle sample from one male Wistar rat was used for this experiment.

Figure 4 displays flat‐topped (A) and Windkessel‐loaded (B, D, E) work‐loops performed at room temperature (24°C) at a stimulation rate of 1 Hz. Different work‐loops were achieved by varying either peripheral resistance, *R_p_* (B), arterial compliance, *C* (D), or characteristic aortic impedance, *Z_C_* (E). Changing *R_p_*, while maintaining *C* and *Z_C_* constant, resulted in a range of work‐loops of different afterloads (B, varying *R_p_* from 50 GPa⋅s⋅m^−3^ to 2 TPa⋅s⋅m^−3^), similar to those achieved by changing afterloads in the “flat‐top” protocol (A), and those produced by modeling simulations (Fig. 3A). The Windkessel‐loaded work‐loops achieved a comparable amount of work (area of the loop), with the peak work being performed at a lower end‐systolic stress than that of the flat‐topped work‐loops, as shown in Figure 4C. In Figure 4D, increasing the arterial compliance alone (15 pm^3^⋅Pa^−1^⋅sec^−1^ to 80 pm^3^⋅Pa^−1^⋅sec^−1^) resulted in negligible change to the work‐loops. In Figure 4E, decreasing the aortic impedance alone (0.5 GPa⋅s⋅m^−3^ to 15 GPa⋅s⋅m^−3^) yielded an increase of force during shortening.

**Figure 5:**
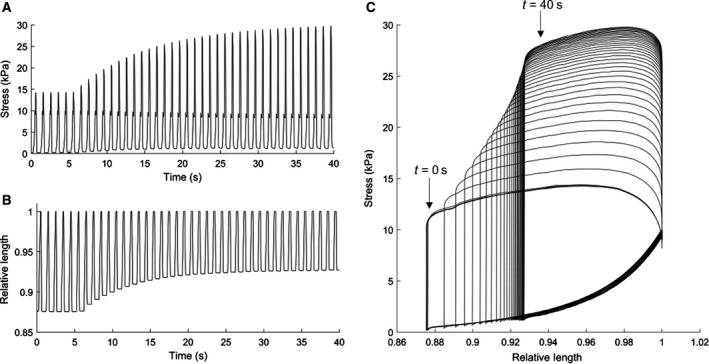
Transient response of stress production (A) and length (B) of a muscle (*L_o_* = 2.47 mm and diameter = 0.259 mm) in response to a sudden change in the peripheral resistance parameter of the Windkessel model. Contraction performed at 1 Hz stimulation and at 32°C. Parameter values *C* = 32 pm^3^⋅Pa^−1^⋅sec^−1^ and *Z_c_* = 5 GPa⋅s⋅m^−3^ were maintained constant. During the time period of *T* = 0 sec to *T* = 6 sec, the peripheral resistance was a low value *R_p_* = 50 GPa⋅s⋅m^−3^. During the diastolic period after the sixth twitch, the value for peripheral resistance was abruptly changed to a high value *R_p_* = 500 GPa⋅s⋅m^−3^. The same transient response of the muscle is also shown in a parametric stress‐length work‐loop domain (C). One muscle sample from one male Wistar rat was used for this experiment.

Figure 5 shows the adaptive response of the muscle to a change in the Windkessel peripheral resistance, *R_p_*. Under subphysiological conditions (32 C and 1 Hz stimulation frequency), *R_P_* was abruptly changed from a low value (50 GPa⋅s⋅m^−3^) to a high value (500 GPa⋅s⋅m^−3^) while the muscle was performing a loaded contraction. This abrupt change of parameter value resulted in a period (40 sec) of transient behavior as both the muscle and the model reacted and reached a new steady state. Figure 5C displays the parametric stress‐length work‐loops during this 40‐second transient period, showing the adaptation of the work‐loops from low to high value of *R_p_*.

**Figure 6:**
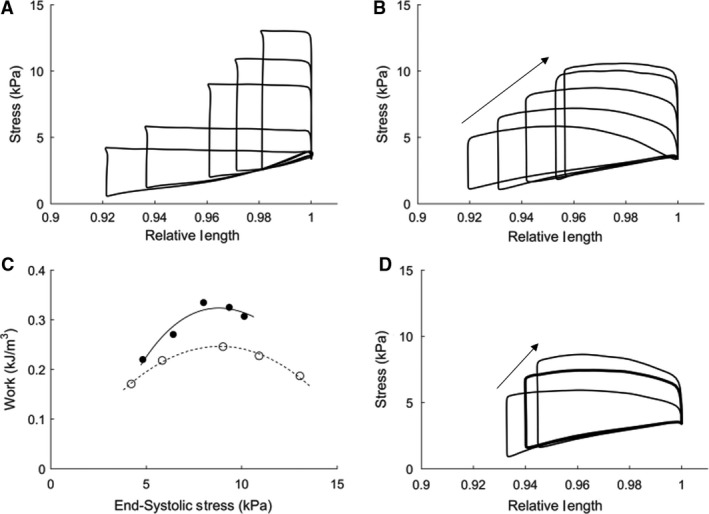
Work‐Loop experiments performed at body temperature (37°C) and 5 Hz stimulation. Trabecula dimensions: *L_o_* = 2.6 mm and diameter = 0.26 mm. Flat‐topped work‐loops of varying constant afterloads (A) and Windkessel work‐loops (B) of increasing peripheral resistance (*R_p_* = 5 GPa⋅s⋅m^−3^ to 40 GPa⋅s⋅m^−3^, as indicated by the arrow). The reference “normotensive” Windkessel parameter values were *R_p_* = 14.5 GPa⋅s⋅m^−3^, *C* = 32 pm^3^⋅Pa^−1^⋅sec^−1^, *Z_C_* = 0.5 GPa⋅s⋅m^−3^. (C) Work output of the muscle during the flat‐topped (unfilled) and Windkessel‐loaded (filled) work‐loops. Polynomial lines of best fit through each dataset are constrained at the origin. (D) Normotensive (bold), hypotensive (*R_p_* = 9.18 GPa⋅s⋅m^−3^, *C* = 42 pm^3^⋅Pa^−1^⋅sec^−1^, *Z_C_* = 0.31 GPa⋅s⋅m^−3^), and hypertensive (*R_p_* = 21.4 GPa⋅s⋅m^−3^, *C* = 15 pm^3^⋅Pa^−1^⋅sec^−1^, *Z_C_* = 0.76 GPa⋅s⋅m^−3^) loads applied to the working trabecula. Arrow denotes parameter change of hypotensive – normotensive – hypertensive. One muscle sample from one male Wistar rat was used for this experiment.

Figure 6 displays flat‐topped (A) and Windkessel‐loaded (B, D) work‐loops at body temperature (37 °C) and at a stimulation frequency of 5 Hz. A reference set of “normotensive” Windkessel parameters (*R_p_* = 14.5 GPa⋅s⋅m^−3^, *C* = 32 pm^3^⋅Pa^−1^⋅sec^−1^, *Z_C_* = 0.5 GPa⋅s⋅m^−3^) determined from the literature was used as a starting point (Fig. 6D, bold). A large range of work‐loops was achieved by varying the peripheral resistance alone from *R_p_* = 5 GPa⋅s⋅m^−3^ to 40 GPa⋅s⋅m^−3^ (B).

In order to model disease conditions, all three Windkessel parameters were changed simultaneously, with respect to the values for normotensive, both hypertensive (*R_p_* = 21.4 GPa⋅s⋅m^−3^, *C* = 15 pm^3^⋅Pa^−1^⋅sec^−1^, *Z_C_* = 0.76 GPa⋅s⋅m^−3^) and hypotensive (*R_p_* = 9.18 GPa⋅s⋅m^−3^, *C* = 42 pm^3^⋅Pa^−1^⋅sec^−1^, *Z_C_* = 0.31 GPa⋅s⋅m^−3^) vasculatures, derived from (Kind et al., 2010). Figure 6D shows that for Windkessel parameters chosen to mimic hypotension (low blood pressure), a lower peak stress was achieved, but a larger extent of shortening occurred. Conversely, for Windkessel parameters chosen to mimic hypertension (high blood pressure), peak stress was increased but with a lower extent of shortening.

## Discussion

A Windkessel–Laplace real‐time model of shortening impedance was successfully developed and applied to isolated trabeculae. The resulting stress‐length work‐loops are thus produced from realistic afterloads that are based on the key parameters describing the mechanical coupling between the aorta and the systemic vasculature. Unlike previous studies, in which muscle tissues were constrained to shorten under a fixed force, this new approach does not explicitly constrain muscle force development during shortening. Instead, the modeled load allows the muscle to traverse a more realistic stress‐length trajectory than previously achieved by fixed‐afterload loading protocols (Figs. 4 and 6). By solving the mathematical model in real‐time, the resulting impedance reflects the appropriate arterial load applicable at any given time‐point, to 50‐µs time resolution. This real‐time loading allows the muscle to adapt dynamically to variations in the arterial impedance load based on changes in model parameters (Fig.5) in a manner that more closely reflects how cardiac performance and arterial impedance interact in vivo. This is the first time a model‐based load has been applied to isolated cardiac tissue in real‐time, without the need to precalculate the impedance load prior to the muscle contraction protocol in question.

This new loading protocol can be readily adopted for future studies aiming to investigate cardiac tissue mechanics under various cardiac pathologies. A key capability of this loading protocol is the ability to parameterize the loads to encompass the active force range of the muscle. Changing peripheral resistance, for example, allows the development of a range of work‐loops which is comparable to that achieved by the previous isotonic loading protocol (Figs. 4 and 6). By comparison, varying the aortic compliance or the characteristic aortic impedance alone does not vary the overall magnitude of the impedance load, shown by the work‐loops depicted in Figure 4D and E. This observation aligns with results observed during whole heart impedance loading experiments with a similar model‐based load (Sunagawa et al., 1985) and simulations carried out prior to experimentation (Fig. 3).

The extension achieved by our approach is the ability to modulate all three Windkessel parameters in order to mimic diseased arterial systems presented to isolated tissues. These “diseased loads,” such as systemic hypertension (Segers et al., 2000) or pulmonary hypertension (Lankhaar et al., 2006; Fukumitsu et al., 2016), can be directly parameterized by measured values, or those obtained from the literature. The “diseased models” can be applied as real‐time loads for studying disease in healthy trabeculae. This is a fundamental improvement over previous studies of hypertension‐induced (Han et al., 2014a; Tran et al., 2016; Han et al., 2017; Pham et al., 2018) or diabetes‐induced (Han et al., 2014b) hypertrophy which have applied isotonic shortening loading protocols with afterloads arbitrarily chosen to span the spectrum of stress from its minimum isotonic value to its maximum isometric value.

In disease conditions, all three Windkessel parameters can vary simultaneously. For example, the development of hypertension is commonly accompanied by an increase in the peripheral resistance and characteristic aortic impedance, but a decrease in the arterial compliance. Our loading protocol not only captures these changes but also facilitates the investigation of the individual effects of each parameter on the disease. Our model simulations and experiments reveal that the peripheral resistance has the most pronounced effect on the load range experienced by the muscle (Figs. 4B and 6B), whereas the remaining two terms primarily affect the curvature of the shortening trajectory (Fig. 4D and E).

The model provides further insight into the mechanism underlying the pronounced effect of peripheral resistance on the work‐loop. Variables such as the onset of shortening, peak stress development, and end‐systolic length are highly dependent on the peripheral resistance, as observed in Figure 5, when this parameter is abruptly changed from a low value to a high value. During shortening, increased values for *R_p_* simulate increased resistance of the peripheral vasculature to blood flow, therefore imposing a higher load on the muscle. This results in a narrowing of the work‐loop (Figs.4B and 6B). The transient response of the muscle adapting to this intervention is readily observable, revealing a progressive increase in stress and reduction in shortening when the load is suddenly increased (Fig.5).

An interesting outcome of applying Windkessel model‐based loading is that, for the muscles used in this study, the mechanical work output of the muscle while experiencing the Windkessel load appears to be higher than that when it is subjected to the force‐constrained loading protocol at the same end‐systolic stress. This observation is more apparent at lower end‐systolic stresses and, although has marginal effect at room temperature (Fig. 4C), is more pronounced at body temperature (Fig. 6C). This observation suggests that previous isotonic loading protocols may have limited the work capability of the muscle and thereby underestimated the mechanical potential of the tissue preparations. However, as the work carried out in this study is only intended to demonstrate the usefulness of our model‐based loading protocol, further experimentation on many more samples would be required to robustly establish whether this effect is significant.

A powerful capability enabled by this system is the ability to enable diseased muscle to experience healthy loads or, vice versa, healthy muscle to experience disease loads (Fig. 6D). Such “cross‐loading” protocols, coupled with Windkessel parameterization to include various sets of “disease parameters,” allow investigation of the range of severity of any given disease model. This cross‐loading capability provides the opportunity to shed light on the origin of experimentally and clinically observed mechanical deficiencies of the myocardium. Although we have implemented a 3‐element Windkessel model in this work, in principle, this approach can allow any mechanical load to be imposed that can be expressed as a *z‐domain* transfer function. This could include the use of a complex characteristic aortic impedance in order to represent wave propagation effects in the aorta.

### Limitations

The loading system is limited by several factors, primarily the employment of the Laplace Law to approximate the ventricle as a thin‐walled sphere. The use of a more complex model of the ventricle, such as a finite element model, could provide a more accurate relationship between volume and length, at the considerable expense of computation time, which would jeopardize the real‐time nature of the loading system. Additionally, the Laplace Law assumes that all myocytes experience the same load in vivo, ignoring any spatial variation present in ventricular tissue. Similarly, control of muscle length during a work‐loop contraction ignores any heterogeneity of sarcomere length within the trabecula.

Secondly, while the use of a 3‐element Windkessel has been shown to adequately represent arterial pressure, the model could be expanded to incorporate more model elements to better describe this complex mechanical system. Previous studies have assessed that the 3‐element Windkessel provides improvement over the 2‐element Windkessel (comprising only a compliance and peripheral resistance), particularly during systole (Frank, 1899; Westerhof et al., 2009). The 4‐element model includes the addition of an inductive term to represent the inertial effects of the movement of blood through the aorta. Although the influence of the inertial element has proven to be minimal, the inclusion of the fourth element could slightly improve the performance of the loading system.

The high speed of model computation allows us to tightly control the aortic impedance load applied to the muscle, and to vary the model parameters in real‐time. In this study, we display the ability of our system to abruptly change parameters of the Windkessel model, in this case the peripheral resistance, and observe the transient changes in force development and muscle shortening as the muscle and model adjust to the change in load impedance (Fig. 5). We also observe the steady‐state behavior of the muscle when contracting against a range of discrete impedance loads (Figs. 4B and 6B) However, in vivo physiological parameters such as those represented by the Windkessel often vary on a beat‐to‐beat basis in response to changes in heart rate, vascular resistance, hormone production, and other physiological conditions. The investigation into how slow changes in Windkessel parameters influence the work potential of cardiac muscle could be highly advantageous, and will be a focus of further study.

### Summary

Our novel implementation of a Windkessel model of arterial characteristics provides a more physiologically realistic, real‐time load for studying isolated cardiac muscle. This new model appears to “unlock” the mechanical work potential of the muscle. This approach is readily applicable to the study of various disease conditions and may shed light on mechanical defects underlying cardiac impairments.

## Conflict of Interest

None declared.

## Appendix A

### Mathematical model formulation

The implementation of the Windkessel model was achieved by encoding the series of equations characterizing the electrical equivalent circuit (Fig. 1B) in a deterministic hardware‐ and software‐based control system. Kirchhoff's current law allows us to express the flow, *Q*, in the resistor *Z_C_* as

(A1) $$Q\left(t\right)=C\frac{\text{d}P_{p}\left(t\right)}{\text{d}t}+\frac{P_{p}\left(t\right)}{R_{p}}$$

while Kirchhoff's second law gives the voltage (pressure) in the peripheral system (*P_p_*) as

(A2) $$P_{p}\left(t\right)=P_{a}\left(t\right)-Q\left(t\right)Z_{C}.$$

Combining Equations 1 and 2 produces the characteristic equation of the 3‐element Windkessel Model

(A3) $$\left(1+\frac{Z_{C}}{R_{p}}\right)Q\left(t\right)+Z_{C}C\frac{\text{d}Q(t)}{\text{d}t}=\frac{P_{a}\left(t\right)}{R_{p}}+C\frac{\text{d}P_{a}\left(t\right)}{\text{d}t}.$$

This time‐domain differential equation was Laplace‐transformed to express the system in the *s‐*domain, where pressure and flow (Eq. 4) are related by the transfer function *Z*_Wk_(s) that describes the overall impedance of the system. Note that Equation 4 is analogous to *V* = IR (Ohm's Law).

(A4) $$P_{a}\left(s\right)=Q\left(s\right)Z_{\text{Wk}}\left(s\right)$$

(A5) $$Z_{\text{Wk}}\left(s\right)=\frac{\left(Z_{C}R_{p}C\right)s+\left(Z_{C}+R_{p}\right)}{\left(R_{p}C\right)s+1}$$

The resulting flow rate out of the ventricle was then transformed back to a speed of shortening by the muscle and applied as a change of muscle length (Fig. 2).
